# A pilot evaluation of the Strengthening a Palliative Approach in Long-Term Care (SPA-LTC) program

**DOI:** 10.1186/s12904-020-00599-w

**Published:** 2020-07-13

**Authors:** Sharon Kaasalainen, Tamara Sussman, Genevieve Thompson, Lynn McCleary, Paulette V. Hunter, Lorraine Venturato, Abigail Wickson-Griffiths, Jenny Ploeg, Deborah Parker, Shane Sinclair, Vanina Dal Bello-Haas, Marie Earl, John J. You, the SPA-LTC team

**Affiliations:** 1 School of Nursing, McMaster University, 1280 Main Street West, HSC 3N25F, Hamilton, ON L8S 4K1 Canada; 2 School of Social Work, McGill University, 3506 University St., Montreal, QC, Canada; 3 College of Nursing, Max Rady Faculty of Health Sciences, University of Manitoba, 89 Curry Place, Winnipeg, MB Canada; 4 Faculty of Applied Health Sciences, Brock University, 1812 Sir Isaac Brock Way, St. Catharines, ON L2N 3A1 Canada; 5 St. Thomas More College, University of Saskatchewan, 1437 College Drive, Saskatoon, SK Canada; 6 Faculty of Nursing, University of Calgary, 2500 University Drive NW, Calgary, AB Canada; 7 Faculty of Nursing, University of Regina, 3737 Wascana Parkway, Regina, SK Canada; 8 Faculty of Health, University of Technology Sydney, 235 Jones St, Ultimo, Australia; 9 School of Rehabilitation Science, McMaster University, 1400 Main Street West, IAHS 403E, Hamilton, ON Canada; 10 School of Physiotherapy, Dalhousie University, 5869 University Avenue, Halifax, NS Canada; 11 Division of General Internal and Hospitalist Medicine, Credit Valley Hospital, Trillium Health Partners, 2200 Eglinton Ave W, Mississauga, ON Canada

**Keywords:** Palliative approach, Palliative care, End-of-life care, Long-term care

## Abstract

**Background:**

Despite increased annual mortality in long-term care (LTC) homes, research has shown that care of dying residents and their families is currently suboptimal in these settings. The purpose of this study was to evaluate resident and family outcomes associated with the Strengthening a Palliative Approach in LTC (SPA-LTC) program, developed to help encourage meaningful end of life discussions and planning.

**Methods:**

The study employs a mixed method design in four LTC homes across Southern Ontario. Data were collected from residents and families of the LTC homes through chart reviews, interviews, and focus groups. Interviews with family who attended a Palliative Care Conference included both closed-ended and open-ended questions.

**Results:**

In total, 39 residents/families agreed to participate in the study. Positive intervention outcomes included a reduction in the proportion of emergency department use at end of life and hospital deaths for those participating in SPA-LTC, improved support for families, and increased family involvement in the care of residents. For families who attended a Palliative Care Conference, both quantitative and qualitative findings revealed that families benefited from attending them. Residents stated that they appreciated learning about a palliative approach to care and being informed about their current status.

**Conclusions:**

The benefits of SPA-LTC for residents and families justify its continued use within LTC. Study results also suggest that certain enhancements of the program could further promote future integration of best practices within a palliative approach to care within the LTC context. However, the generalizability of these results across LTC homes in different regions and countries is limited given the small sample size.

## Background

As the population ages, more people will die in long-term care (LTC) homes. In Canada, annual mortality rates of residents in LTC range from 27 to 52.3% [1]. Similar trends have been noted in other countries including the United States [2], the United Kingdom [3], and Australia [4]. Despite these trends, care of dying residents and their families continues to be suboptimal in LTC; with pain and other symptoms being poorly managed, especially for those with dementia [5]; lack of attention to advance care planning (ACP) [6] and issues of loss, grief and bereavement [7]; widespread use of feeding tubes [8]; and excessive reliance on hospitalizations [9].

In response, a number of initiatives have been implemented to improve the quality of living and dying for LTC residents with a life-limiting illness and their families [10–13]. Features that appear to support effective and sustained palliative care implementation and show some promise in improving care delivery include: (a) mechanisms that allow for the assessment and identification of gaps in current practices and philosophies, (b) mechanisms to help staff identify and activate a change in care planning based on key transition points, (c) formalized opportunities for communication between staff, residents and families, and (d) team-building strategies, champions or resource teams and collaborative learning opportunities.

**Fig. 1:**
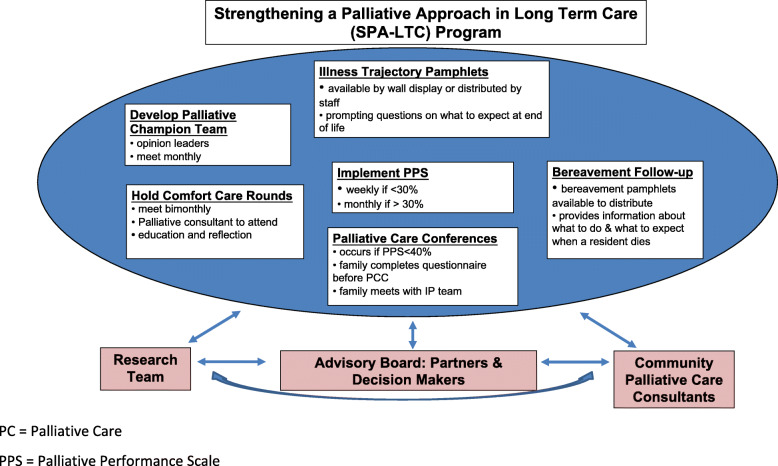
The Strengthening a Palliative Approach in Long Term Care (SPA-LTC) Program

Addressing these barriers and building on the growing evidence aimed at improving identification, communication and capacity; our team developed a multi-component program, called Strengthening a Palliative Approach in LTC (SPA-LTC; Fig. 1). It is consistent with the SPA-LTC model, that was developed based on a scoping review [13] of the literature and stakeholder analysis [14]. As such, the SPA-LTC program consists of the following **core, evidence-informed components:** (a) an interdisciplinary palliative champion team (to provide leadership and support implementation); (b) condition-specific pamphlets (to provide information about condition-specific end of life trajectories to residents and families) [15, 16], (c) the Palliative Performance Scale (PPS) (to trigger end of life discussions) [17]; (d) Palliative Care Conferences (PCCs) (to provide a forum for family communication about end-of life preferences and needs) [18]; (e) Comfort Care Rounds (to support peer education, team building and case discussions) [19]; and (f) post-bereavement follow up (to offer families grief support and links to community services) [20].

To support implementation and sustainability in the real world, the program was refined in collaboration with study sites and leveraged existing palliative resources outside of the LTC home sector to provide guidance to LTC staff [14]. For example, in the original iteration of our program we had conceptualized encouraging early communication on future end of life through care conferences offered to stable residents within 6 weeks of relocations and annually thereafter. However, our pre-implementation consultations with staff, residents and families revealed uncertainty regarding the feasibility of such discussions and hence we developed condition-specific pamphlets instead.

The **goal of this study** was to evaluate the impact of the SPA-LTC program on residents and families. Specifically, this study addressed the following research questions:

1. Does the SPA-LTC program improve hospital use at end of life for LTC residents?
2. What are the perceptions of the SPA-LTC program for residents and family members?

## Methods

This study used a convergent, parallel mixed method (quan + QUAL) design to address both of our research questions [21, 22]. Mixed methods research allows researchers to address complicated research questions and collect a richer and stronger array of evidence than can be accomplished by any single method alone [22]. We integrated the qualitative and quantitative data after each component had been analyzed separately using the qualitative data to help complement and elaborate on the quantitative data (i.e., hospital use data) specifically.

Data was collected at baseline (quan) and after the 18-month intervention period (quan + QUAL). Ethical approval was obtained from two universities (McMaster University: #14–863; McGill University: #281–1214).

### Settings

We selected four separate LTC homes in southern Ontario which represented the mix of contexts found in LTC homes across Canada including for-profit; not-for-profit; large (150 beds), medium (100–150 beds) and small (less than 100 beds); high turnover and low turnover; religious-based vs secular [23]. Table 1 shows a comparison of these sites. These conditions have also been found to impact the successful implementation and adoption of change efforts, with homes that are smaller, not-for-profit, and have low turnover rates identified as having better conditions to support change [24].

**Table 1:** Comparison of Sites

Site	Profit/Not-for-profit	# of beds	Administrator Turnover rate	Diversity
**1**	For-profit	206	Moderate	Culturally diverse, various faiths
**2**	For-profit	169	High	Culturally diverse
**3**	For-profit	64	Moderate	Many residents without family/once homeless
**4**	Not-for-profit	112	Low	Operates within the context of Jewish culture and values

### SPA-LTC program

Prior to implementation of the SPA-LTC program, each site was invited to identify staff from interdisciplinary backgrounds to comprise a team of **champion leaders** who would receive **training** on best practices in a palliative approach to care, learn about the components of the SPA-LTC program and oversee implementation in study sites. Former work in the areas of culture change in LTC suggest this type of infrastructure holds promise in supporting implementation and sustainability [25, 26]. Once teams were established, each site was asked to conduct monthly **comfort care rounds** which comprised of case-based learning and exchange about topics of high importance to LTC staff [19]. Palliative experts outside of the LTC sector facilitated these rounds and offered consultation services. Over time some sites used internal leaders to continue facilitating the rounds.

Participating residents and families were invited by staff to attend a **Palliative Care Conference (PCC)**. The PCC component of the SPA-LTC program was adapted from the Palliative Approach Toolkit designed by Dr. Parker, an international co-investigator [12]. The Palliative Approach Toolkit has been implemented nationally in Australian LTC homes and supports a palliative approach in this context. Initially, we held a ‘mock’ PCC with staff to provide specific training and education about approaches and tips about what to expect and ways to deal with challenging situations. Together, staff reviewed the Guide to Conducting Palliative Care Conferences as a precursor to holding one and watched the training video, “On the Same Page”. Documentation guides (Staff Planning Guide, Physician Fax Sheet, Good Palliative Care Plan, PCC Summary; all available upon request) from the Palliative Approach Toolkit were adapted for this study based on local contexts and to ensure they were consistent with the jurisdictional legal framework.

**Condition-specific pamphlets** for five conditions of high prevalence in LTC were made available in participating LTC home via display boards of through staff distribution [15, 16]. Because these resources evolved from feedback received during the course of the project, no formalized protocol for administration was developed. Staff were instructed to consider distributing the pamphlets to those residents and families scheduled for a PCC. Likewise, **bereavement pamphlets** were developed and distributed to family members as directed by staff [20].

### Recruitment of residents to participate in the SPA-LTC program

Following a period of training, designated staff located in each participating LTC home completed the **Palliative Performance Scale (PPS)** for all residents. The PPS was used to screen participants for potential involvement because it provides a framework for measuring progressive decline in palliative residents [17]. PPS scores can be divided into three stages: stable, 100–70%, transition, 60–40%, and end of life, 30% and less. There is evidence that the PPS produces valid, reliable measurement of progressive functional decline in a patient suffering from a terminal or incurable illness. Based on previous pilot work, guidelines have been developed for successful implementation of the PPS in LTC [17].

Residents who were English-speaking and scored 40% or less on the PPS, and/or their family were invited by staff to learn more about the SPA-LTC program and consider participating in research. All potential participants were contacted by a member of the research team who reviewed the research study with them and their potential involvement. Written consent was obtained from the resident and/or family member.

### Data collection and analysis

#### Demographics of participating residents

At baseline, administrative data (quantitative) was collected retrospectively for all residents who had died over the past year. The same data was collected at study end, 18-months later, for participating residents only. Data were collected for the following indicators: (a) number of resident deaths; (b) number of resident deaths that occurred at the hospital versus LTC home; and (c) number of residents who visited the emergency department visits in their last year of life. Relative risk reduction (RRR) and confidence intervals (CIs) were calculated for the proportion of resident deaths that occurred in hospital and emergency department use comparing the baseline (all residents) and post-implementation (participating residents only) data.

Chart reviews of participating residents also included demographic information, admission information (date and diagnosis), Charlson Comorbidity Index (a measure that aims to categorize comorbid medical conditions that can alter mortality risk) [27], PPS scores and palliative care conference notes. Chart data were analyzed using descriptive statistics (i.e., frequencies, percentages) across all four sites at both time-points (i.e., before and after implementation).

### Perceptions of the SPA-LTC program

#### Family members

At study end all bereaved family members of enrolled residents were invited to participate in telephone interviews to capture their perceptions and experiences of the SPA-LTC program, with particular focus on attending a PCC. For families who attended a PCC, we asked them to report on an additional four closed-ended questions, using a 10-point Likert scale, on the following items: (a) how helpful was the PCC, (b) how supported did you feel by staff during the PCC, (c) how well were your concerns addressed at the PCC, and (d) how comfortable did you feel making arrangements or decisions after the PCC. Next, we continued the interview with some qualitative questions that probed them to describe their perceptions about these topic areas as well.

For bereaved family members who did not attend a PCC, we asked them about: (a) the circumstances surrounding the death of their family member and how the LTC staff supported them, (b) their experiences at the LTC home with end of life care at the LTC home, and (c) what recommendations they had for LTC homes to better support residents and families at end of life. Qualitative data were transcribed and coded using thematic content analysis [28]. Specifically, important concepts that emerged from the data were labeled, categorized, and coded. Initially, two individuals coded the transcripts independently. Discrepancies were discussed among the team until consensus on a coding framework was reached.

#### Residents

Unfortunately, there were no residents who were able to participate in an interview at the end of the study or after having attended a PCC due their extremely frail condition or death. However, we were able to conduct a focus group with other residents to gather their perspective about other components of the SPA-LTC program that were implemented in their LTC home.

Four focus groups were held with residents (one focus group per LTC home). Residents were asked about their general perceptions about the care provided for residents who were dying in general and about the SPA-LTC program in particular (if able). Focus group data were analyzed in a similar fashion as the family interviews, and in addition, we paid particular attention to exploring interaction among residents in their discussion. In this manner, we were able to highlight areas where residents agreed upon on ideas discussed or built on one another’s discussion points [29].

## Results

### Characteristics of residents who participated in the SPA-LTC program

**Fig. 2:**
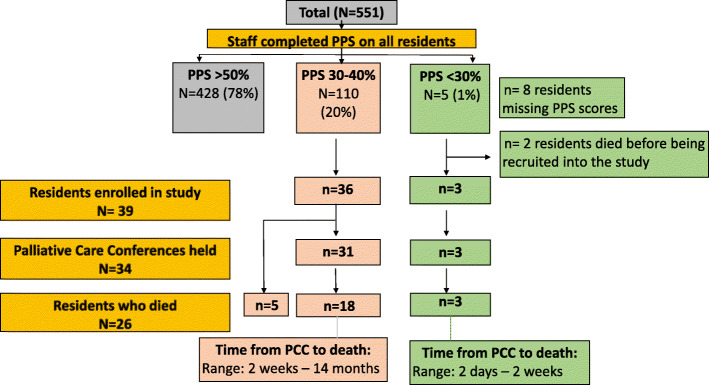
Participant Flow Diagram

The total number of residents living within the four participating LTC homes was 551, and staff completed PPS scores for 99% (*n* = 543) (Fig. 2). Of these residents, 20% had a PPS score between 30 and 40% (*n* = 110) and 1% (n = 5) had a PPS score less than 30%. Of the 115 eligible participants 39 residents/families agreed to participate in the study representing a response rate of 34%. Of the 39 participating residents, 23 (59%) were female, and 34 (87%) had a diagnosis of dementia. The average Charlson Comorbidity Index score was 7.13 (Table 2).

**Table 2:** Characteristics of Participating Residents (*N* = 39)

Characteristic	n (%)	Mean (SD)^a^
Sex		
Male	16 (41)	
Female	23 (59)	
Age at the time of enrolment (years)		84.6 (10.9)
Length of Stay in LTC (years)		5.6 (3.3)
Charlson Comorbidity Index		7.1 (2.00)
Palliative Performance Scale		
• < 30%	3 (7.7)	
• 30–40%	36 (92.3)	

^a^Standard Deviation

### Hospital use at end of life for residents

Baseline chart audits revealed that 25.6% (141/551) of all LTC residents living within the four participating homes died in the year prior to study implementation, with the majority of the 141 resident deaths (71.6%) occurring at the LTC home (Table 3). Of the 141 residents who died, 92(65.3%) of them visited emergency departments during the last year of life.

**Table 3:** Site of Death and Emergency Department (ED) Visits in the Last Year of Life at Baseline (All Residents) and Post Implementation (Participating Residents Only)

OUTCOME	Pre ***n*** = 141 n (%)	Post ***n*** = 26 n (%)	RRR^a^	Confidence Intervals
**Had PCC before Death**	N/A	21 (80.7)	–	–
**Hospital Deaths**	40 (28.4)	2 (7.7)	72%	0.05, 0.93
**ED Visits**	92 (65.3)	8 (30.8)	54%	0.15, 0.73

Notes: ^a^*RRR* Relative Risk Reduction

At study end, the same data were collected for participating residents only. Of them, 87.2% (34/39) had a PCC during our 18-month data collection period (Fig. 2), with 80.7% (21/26) having a PCC before they died (Table 3). Findings indicate that there were statistically significant reductions in rates of hospital deaths (RRR: 72%, CIs: 0.05, 0.93) and emergency department visits (RRR: 54%, CIs: 0.0.15, 0.73) during the last year of life for residents who participated in the SPA-LTC program.

### Perceptions of the SPA-LTC program

#### Family members

In total, 19 bereaved family members agreed to be interviewed; 14 of them attended a PCC and 5 did not attend a PCC before they died. Since the PCCs were the most visible component of the SPA-LTC program for residents and families, interviews focused predominantly on experiences with PCCs and overall experiences with end of life care.

##### Attended PCC

For the closed-ended questions in the interview, family members who attended a PCC rated it as useful (Mean (M): 9/10; Standard Deviation (SD): 1.2), (b) they felt well supported at the PCC by staff (M: 8.9/10; SD: 1.1), (c) their end of life concerns were addressed well (M: 8.7/10; SD: 1.4), and (d) they were comfortable with their decisions about end of life care after the PCC (M: 8.7/10; SD: 1.5). See Table 4.

**Table 4:** Responses of Families who Attended a Palliative Care Conference (N = 14)

Question^a^	Mean (SD)^b^
1. How helpful was the Palliative Care Conference (PCC)?	9.0 (1.2)
2. How supported did you feel by staff during the PCC?	8.9 (1.1)
3. How well were your concerns about your friend/relative’s end-of-life care addressed at the PCC?	8.7 (1.4)
4. How comfortable did you feel about making end-of-life arrangements or decisions after the PCC?	8.7 (1.5)

^a^Likert scale ranged from 0 (not at all) to 10 (a great deal)

^b^Standard Deviation

Qualitative findings from the interviews emerged across three main themes. Families described how attending a PCC: (a) helped them feel ‘all on the same page’; (b) promoted individualized, holistic care; and, (c) enabled information-sharing and effective communication between LTC staff and families to be better prepared.

##### ‘All on the Same Page’

The majority of family members reported that their concerns and the kind of care and services wished for at end of life were addressed at the meeting and that they felt better afterwards than before it. The PCCs encouraged discussion that helped inform decision-making so families and staff were in agreement and had a mutual understanding of plans moving forward. One family member stated,

*The quality of my experience was great. I feel like everybody knew what was going to happen when it started to happen, and there’s comfort in that, and what could have been a terrible and difficult time was less terrible and difficult because we all knew what she wanted* (Site 4, FM 12, no ED visit, died in LTC home).

##### Individualized, holistic care

Findings from the family interviews highlighted that attending a PCC promoted individualized and holistic care for the resident. For example, a family member described how the PCC allowed her to outline the kind of care and services that her mother (resident) would want at the end of her life, including religious activities:

*We’re Catholic, so the priest to came over one or 2 days before my mother passed.. to give her the last rite, and that was of extreme comfort to me.. it was just all those prong … it was the effort that the director of care took to notify all the people concerned with the end of life care. The social worker even gave me e-mail addresses of people who could help me as counsellors, after my mom died, which was wonderful (Site 1, FM1, no ED visit, resident died in LTC home).*

When probed to respond about how their family member’s care had changed after the PCC, one participant stated,

*My mom used to take all kinds of vitamins and so on, they removed all of that, because she was regurgitating, so that helped* (Site 1, FM5, had ED visit, died in LTC home).

Although attending a PCC helped promote individualized care for the resident, there was some concern that individualized care for families once the resident dies is needed. A family member emphasized the importance of supporting families who may be struggling, especially during the bereavement phase:

*If the family members need grief counselling … because at times I wondered where would you go for that sort of thing if it becomes necessary … I mean the death is expected when someone’s old but you don’t know it’s gonna affect you right?* (Site 3, FM4, no ED visit, died in LTC).

##### Improved communication

The majority of family members who attended a PCC stated that communication between families and staff had improved and that they felt better informed and better prepared after having attended the PCC. For example, one family member stated,

*Do whatever they can to make him comfortable, that’s what my priority would be. Also letting me know what’s going on … basically just giving me the information I needed for him, like being kept up-to-date (Site 3, FM9, no ER visit, died in LTC home).*

Another family member stated:

*I really appreciated that I could make preparations prior to her passing, rather than waiting so that I’m not under a rush when I have to do it … .you don’t have much time to make these decisions, and if it’s your first time, its not an easy thing … so it’s preparing …* (Site 2, FM8, no ED visit, died in LTC home).

Families acknowledged that having conversations to prepare them for what to expect was not easy, but still they felt that it was worth it so they could feel better prepared when the time came for them to have to make decisions about end of life care for their loved one.

#### Did not attend PCC

For family members who did not attend a PCC, we found divergent findings from those who attended a PCC. The main themes that emerged from these interviews were: (a) feeling that a PCC was not needed, (b) wishing for better communication, (c) needing more information to know what to expect ‘down the road’.

##### Family felt PCC was not needed

When probed, most family members stated that they felt that a PCC was not necessary or needed at the time for different reasons. One family member stated that she had enough regular contact with the LTC staff that she felt she didn’t need an additional PCC:

*My mother was there for 3 years, the nurse there who knew me well sat me down and kept me in touch constantly with the physician, and I was able to contact the physician any time (Site 4, FM6, no ED visit, died in LTC).*

Another family member felt she had the expertise and was prepared to make decisions on behalf of the resident due to previous advance care planning conversations with the resident:

*It [a PCC] wasn’t necessary because I’m a nurse myself, and I knew that he had decided that this was what he wanted, and I told them I didn’t want any IV’s, I didn’t want any life supports.. And I went there everyday, so I was on top of everything (Site 4, FM14, no ED visit, died in LTC home).*

##### Wishing for better communication

Family members highlighted the need for better communication with LTC staff but that this was generally not occurring in their experiences.

*I guess better communication, I found that it was kind of sparse, and I didn’t always find that observations were really getting communicated amongst the team members, but I would bring things forward that I noticed, and I kind of wished that they had been more in tune to maybe bring things forward to me, or thoughts or observations (Site 4, FM11, had ED visit, died in LTC home).*

The need for communication was even more pronounced when the resident had challenges with communicating with LTC staff. In this case, families became even more reliant on staff to help inform them of current care issues and care planning. One family member describes,

*I don’t know in that sense, it’s more – in his situation it was really about hearing, he didn’t hear very well and that was really the main problem with his quality of life is that he didn’t understand people and people didn’t communicate with him because it was very difficult, so at end of life just communication was really important, because there was great uncertainty about what was going on and what treatments he was receiving, and being informed about decision-making as well.. so I think mainly just around communication. (Site 2, FM7, had ED visit, died in LTC home).*

##### Needing more information to feel prepared

Many family members spoke about how poor communication with staff led to their need for more information to help prepare them for decisions to make as their loved one was nearing end of life. One family member stated,

*I think being aware of the stages is really important for family members.. if I had known more of the stages, I could have planned my time better to be with my mother, and for her.. I really needed somebody else to tell me, especially from an emotional standpoint, that she is deteriorating now and I think it would have been helpful for someone to really sit me down, and to think about, to digest, and to come to terms with what’s ahead …*. *I wish somebody could have told me more – a lot of the time I was getting phone calls – I suspect people [at the facility] were using cell phones, and I could barely hear what they’re saying –and so I’m saying “pardon me, pardon me, pardon me,” and I can tell that they’re getting frustrated, because they just want to make the call and go home, it was the end of their shift, and I’m sorry, but if you can’t articulate what’s really important to me, don’t waste your time or mine (Site 4, F6, no ED visit, died in LTC home).*

Family members stated that they would have appreciated discussing end of life topics with staff so that they would feel more prepared when the time comes, even if the discussions were difficult. A family member comments:

*And they seem reluctant to sort of – like, my mom was old with worsening dementia right, like the funeral arrangements, I don’t know, they didn’t seem to want to like broach that subject, but they should … I needed to know what to do, if I happened to be out of the country on vacation or on business or whatever, right? (Site 3, FM3, had ED visit and died in hospital).*

Family members stated that having the information ahead of time would have helped to make informed decisions at end of life given the pressing nature in which these decisions need to made. A family member stated,

*You don’t have much time to make all these decisions, and if it’s your first time, it’s not an easy thing, I found that out with my mother. So it’s – it’s preparing (Site 4, FM11, had DR visit, died in LTC home).*

### Residents

In total, 36 residents attended four focus groups (one per site), with the number of participants in each focus group ranging from 8 to 11 participants. Residents’ average age was 69.9 (SD: 11.5) with most being female (58.3%) and having lived in the LTC home for under 5 years (63.9%).

Analysis of the resident focus groups revealed four main themes: (a) learning about a palliative approach, (b) wanting to be informed about their current status and information about their condition, and (c) promoting a dignified and person-centered approach for dying residents.

#### Learning about a palliative approach

During the focus groups, there was some discussion among residents about how the SPA-LTC program has broadened their understanding of a palliative approach to care beyond the traditional view of simple end of life care. In particular, one group of residents discussed their appreciation of learning about a palliative approach to care by reading one of the study pamphlets:

*Person (P)1: Yeah, I like the palliative approach because before I came here palliative approach meant you were dying …*

*Interviewer (I): Right.*

*P4: … Okay and when they mentioned palliative approach I thought, ahhh … you know.*

*I: You automatically think end of life.*

*P7: Yeah end of life but it’s not so.*

*P7: Because people can be in palliative care for 5 years.*

*I: Right.*

*P1: I always thought too that palliative care was the next thing to death.*

*P7: Yeah.*

*P1: And just as D [another resident] said here it’s a long-term treatment.*

*P3: As long as it doesn’t mean totally mean giving up on someone … (Site 1).*

However, residents commented on the need for staff to receive more education about implementing a palliative approach to care. In fact, two residents felt that education should be mandatory:

*P1: I think one of the big things is that the staff need to be educated.*

*P4: Yeah but even though we are in this pilot project I find staff still … a lot of them … instead of it [staff education] being a choice, I think they should be made to.*

*I: Like have it mandatory for all of the staff?*

*P4: Yes, mandatory, yes (Site 3).*

##### Wanting to be informed

Residents highlighted the importance of having as much information and as early as possible, even right at admission, so they are informed about what to expect. A resident commented:

*I don’t think it’s right for the doctor to keep things from you.. well usually a doctor tells you. It’s better for them to tell you, yourself … you don’t want to be kept out of it (Site 2).*

One resident stated that having an accessible computer to retrieve information while they were still able to understand it would be very helpful.

*As you go on you may be diminished in your capacity to do things but in that, going onto that period it still would be nice as a resident here if we could have access to a good computer so you could find out information and still be aware of what’s going on with their illness (Site 1).*

According to one resident, the importance that family is informed was discussed, even if they are not receptive to talking about it. A resident describes:

*I’ve already talked with my family about this and that, but you know, what I’ve decided is same as when my husband died, we both agreed that they [family members] know about it but they just don’t want to believe, you know, young people don’t want to believe anything like that (Site 3).*

##### Importance of a person-centered approach to care of the dying

Many residents agreed that they have seen improvements in the way care for the dying has been implemented over the intervention period. One resident stated:

*I like the idea that- it is really providing a whole new approach to looking at the wonderful things that go on here and I hadn’t really been totally aware of all of it but if all of this is being followed here, which I believe it is, then this is superb. (Site 1).*

However one resident described some concerns about the lack of individualized spiritual care, stating:

*I know in my country they used to call the priest all the time. Here you don’t know what to expect. If I’m going I don’t get special care (Site 4).*

The discussion in another focus group highlighted the need for privacy to provide a comfortable environment for residents, including roommates, and families when a resident was dying:

*P4: But the thing is you don’t have a room. So if you have two people in a room and one person is in palliative care and the family is in and out and they have to stand in the hall and they can’t sit down and have a cup of coffee, or just sit down and catch their breath, it’s very hard to be with the person in palliative care.*

*P3: It’s difficult for the roommate of the person who is palliative because you don’t get any rest. You don’t get any privacy. So it has been my experience that when this happens I just leave but you can’t do that in the middle of the night.*

*I: Of course.*

*P3: You can do it in the daytime but you can’t do it at night and so it can be very disruptive but you just, you get no peace or relaxation.*

*I: Okay.*

*P3: You can’t put it in the room, but they are terrible to sit in anyway. When my roommate was dying I had to get out of the room and try to get some sleep so fine I will take a pillow and a blanket I will go sit in one of those chairs out front and I lasted less than five minutes. It was so uncomfortable I went back to my bed. I said I can’t stand this (Site 3).*

## Discussion

These study findings support the SPA-LTC program. Across four LTC sites, this program reduced hospital use at end of life, with significant reductions in emergency department use and hospital deaths. Both the quantitative and qualitative data from the family interviews indicated that families experienced PCCs positively. That is, they reported that a PCC was helpful and provided support to them, and that they felt comfortable making end-of-life decisions for residents after attending a PCC through the closed-ended questions. The open-ended questions or qualitative findings elaborated on these responses by describing how the PCCs improved communication with health care providers and allowed them to feel “all on the same page”. Given these findings, it is quite likely that by attending a PCC, families were able to address their questions and concerns and become informed so that they could feel more comfortable making decisions about end-of-life issues which contributed to the reductions in emergency department use and hospital death, allowing residents to remain in LTC until their death.

Other research also supports the effectiveness of multidisciplinary team meetings (such as PCCs) that include family members and residents (if able), to reduce hospital use for LTC residents [30–32]. For example, Phillips et al. (2013) found that PCCs improve medication management and support a palliative approach to care for people with advanced dementia in LTC [33]. However, they stated that PCCs are feasible if the identified barriers are addressed and the facilitators optimized, namely related to the capacity of physicians to contribute to the interdisciplinary care planning. Certainly, the demands placed on staff to organize and hold PCCs is important to consider. Perhaps optimizing already scheduled annual care conferences in LTC could help offset the demands of holding an additional PCC. To do this, the annual care conferences would need to be adapted to include more emphasis on ACP and goals of care discussions, which generally is not the case. Clearly, a shift in culture is needed to fully integrate a palliative approach to care.

Interestingly, residents who participated in this study commented that they appreciated new learning about a palliative approach to care which seemed to resonate well with them and their needs. A palliative approach incorporates advance care planning which aims to begin conversations earlier about values and wishes that inform goals of care decisions later at end of life. In doing so, these conversations may be less ‘depressing’ or stigmatized; ultimately helping prepare families and residents while minimizing the stress and guilt about making these critical decisions when the need arises.

Research suggests that the absence of ACP contributes to higher rates of hospital transfers of LTC residents [33]. As such, the need for ACP to be regularly occurring in LTC is increasingly recognized [34, 35]. LTC residents and their families have reported that more preparation and information helps them make critical decisions later on when end of life is near [34], which is consistent with our study findings. This is even more important for those residents who have cognitive impairment, who have limited time that they can engage in ACP discussions with their family or LTC staff. There is a need for ACP documentation so staff at palliative care conferences can reflect on previous discussions so that goals of care at end of life can be developed with residents/families/substitute decision makers.

Clearly, it is important to engage families and residents in advance care planning discussions help to empower them to make decisions that align with residents’ preferences and wishes and receive goal-concordant care at the end of a resident’s life. This is important to residents, as our study findings suggest, and often spans beyond the bio-medical components of care. According to Bökberg et al., in order to improve the quality of life for residents living in long term care, the focus of care needs to be person-centered and based on a holistic approach [36]. In fact, one could argue that a holistic approach becomes even more important when someone is dying in LTC when providing comfort care is paramount. As such, efforts should be directed at supporting residents to die in their home, as opposed to being transferred to the hospital during the last year of their life, if this is their wish.

Another strategy that is emerging as a potential benefit to LTC homes to help avoid emergency department transfers at end of life is the community paramedic outreach program. This involves paramedics receiving additional training in: (1) geriatric assessments and management, (2) end of life care, (3) primary wound closure techniques, and (4) point of care testing [37]. Medical oversight is provided by a physician along with guidelines, available to LTC homes between 9 am and 9 pm, 7 days a week. Paramedics are dispatched by the communication center when requested by LTC staff and must communicate with either the overseeing physician or the LTC physician at each visit to make decisions about plans for care [37].

### Strengths and limitations

There were strengths and limitations to this survey. First, this study used a pre-post study design with no control group so our only control measure was our baseline scores, which could have accounted for some bias in our sample. Also, we were not able to compare the characteristics of our baseline sample of residents with the participating (post) resident sample so we could not assess if the two samples were comparable for our analysis. We were not able to interview residents who engaged in PCCs as they were too frail and most had advanced cognitive impairments so we had to rely on their family members to gather information about the PCC.

Finally, our small response rate of family members who agreed to be interviewed so interpretation of the acceptability of the SPA-LTC should be taken with caution. Finally, the generalizability of these results across LTC homes in different regions and countries is limited given the small sample size.

## Conclusions

Despite the study limitation, this study does provide support for the SPA-LTC program in improving hospital use at end of life and improving family and resident satisfaction with care. Study results also suggest that certain enhancements of the program could further promote future integration of best practices within a palliative approach to care within the LTC context. Future work is needed to evaluate the SPA-LTC program using a randomized control trial with larger sample sizes to provide stronger support for its use and spread in other LTC homes.

## Data Availability

All data generated or analysed during this study are included in this published article. The datasets generated and/or analysed during the current study are not publicly available due to constraints of our ethical review approvals related to privacy laws.

## Abbreviations

LTC: Long term care
SPA-LTC: Strengthening a Palliative Approach in Long Term Care
PPS: Palliative Performance Scale
PCC: Palliative Care Conferences
SD: Standard deviation
RRR: Relative risk reduction
CI: Confidence intervals
ACP: Advance care planning
